# Time-of-Flow Distributions in Discrete Quantum Systems: From Operational Protocols to Quantum Speed Limits

**DOI:** 10.3390/e27100996

**Published:** 2025-09-24

**Authors:** Mathieu Beau

**Affiliations:** Department of Physics, University of Massachusetts, Boston, MA 02125, USA; mathieu.beau.89@gmail.com

**Keywords:** time in quantum mechanics, operational protocols, quantum control, quantum speed limit, time-energy uncertainty, time distribution, shortcut to adiabaticity, open quantum systems, time-dependent Hamiltonians, optimization

## Abstract

We propose a general and experimentally accessible framework to quantify transition timing in discrete quantum systems via the time-of-flow (TF) distribution. Defined from the rate of population change in a target state, the TF distribution can be reconstructed through repeated projective measurements at discrete times on independently prepared systems, thus avoiding Zeno inhibition. In monotonic regimes, it admits a clear interpretation as a time-of-arrival (TOA) or time-of-departure (TOD) distribution. We apply this approach to optimize time-dependent Hamiltonians, analyze shortcut-to-adiabaticity (STA) protocols, study non-adiabatic features in the dynamics of a three-level time-dependent detuning model, and derive a transition-based quantum speed limit (TF-QSL) for both closed and open quantum systems. We also establish a lower bound on temporal uncertainty and examine decoherence effects, demonstrating the versatility of the TF framework for quantum control and diagnostics. This method provides both a conceptual tool and an experimental protocol for probing and engineering quantum dynamics in discrete-state platforms.

## 1. Introduction

Time plays a peculiar role in quantum mechanics: while space is associated with self-adjoint operators and measurable observables, time remains an external parameter. This fundamental asymmetry leads to challenges in defining meaningful *time-of-arrival* (TOA) distributions, especially when compared to the clear probabilistic framework for position measurements [1]. Numerous approaches have been proposed for continuous systems (see reviews [2,3]), yet a consensus remains elusive due to issues such as ambiguity in measurement interpretation, non-uniqueness, and divergence in limiting cases. In contrast, the case of *discrete quantum systems*, relevant to qubit-based platforms, spin chains, and trapped ions, has received far less attention. Proposals such as the Page and Wootters’ mechanism [4] or the quantum clock approach, [5,6], where time is inferred from correlations between a system and an auxiliary quantum clock, are, in principle, applicable to discrete systems. Despite their conceptual depth and promising implications, none of these proposals have yet been explored experimentally in the laboratory. Beyond being a purely conceptual problem, the notion of time is also crucial in modern quantum technologies, in particular for optimal control and decoherence, which both have great importance in quantum computing [7], quantum control [8], quantum simulation [9], quantum metrology [10,11,12], shortcut to adiabaticity (STA) [13,14,15], and finite-time quantum thermodynamics [16].

Different approaches offer a variety of methods for the quantification of time, which can be used to help quantum technology speed up processes. Quantum speed limits (QSLs) [17,18] are useful concepts that tell us how fast a transition could happen and have been used for optimal control [19], decoherence [20], and quantum metrology [6,10]. However, while QSLs offer lower bounds on transition times, they do not provide experimentally accessible information about the time distribution of the transition. Transition timing in discrete quantum systems has been approached through quantum trajectories [7], which model dynamics under continuous monitoring by unraveling the evolution into stochastic jumps or diffusive paths. While this framework captures decoherence and measurement backaction, it requires open-system modeling and continuous observations, which can induce a quantum Zeno effect. Alternatively, first-detection protocols [21] define transition times as the earliest successful projective measurement following stroboscopic sampling. This approach yields useful timing statistics but focuses on first-event detection and relies on invasive monitoring or collapse-based dynamics, limiting their generality and experimental accessibility in closed or control-oriented settings.

As seen above, the passage of time in discrete quantum systems lacks a unifying framework that addresses both theoretical and experimental challenges. Such a framework should not only clarify conceptual issues, such as the time-of-arrival problem, but also support practical, yet fundamental, tasks in quantum engineering. In this work, we propose a general and operationally meaningful concept of *time-of-flow (TF) distribution* to bridge the gap between these complementary aspects. The TF distribution is defined from the absolute value of the time derivative of the probability to occupy a target state and is experimentally reconstructible via projective measurements at discrete time steps on independently prepared systems, thus *bypassing the quantum Zeno effect* for continuous measurement protocols [22]. In monotonic regimes, this TF distribution is interpreted as the time-of-arrival (TOA) or time-of-departure (TOD) profiles and yields expected results in limiting cases (delta pulse model). Beyond conceptual consistency, we demonstrate how the TF distribution provides a practical framework to extract meaningful timing statistics. We apply this approach to optimize shortcuts to adiabaticity (STA), and to analyze *decoherence dynamics* in open quantum systems described by Lindblad-type master equations. This framework, therefore, offers both a theoretical tool and an experimental protocol to explore quantum timing in discrete-state systems. In addition, we derive a TF-based quantum speed limit (TF-QSL) and a new uncertainty relation linking the temporal spread of transitions to their energetic or dynamical generators. These results position the TF distribution as both a practical diagnostic and a fundamental tool for quantum dynamics in discrete systems.

## 2. Heuristic, Empirical and Theoretical Definition of the Time-of-Flow for Discrete Quantum Systems

Heuristically, the time-of-flow (TF) distribution quantifies *how the probability of being in a given quantum state changes in time*. Operationally, it describes the *rate at which probability “flows into or out of” a chosen state* |k〉 during the system’s evolution. Unlike the standard occupation probability $p_{k}\left(t\right)$, which tells us *how likely* the system is to be in |k〉 at time *t*, the TF distribution $\pi_{k}\left(t\right)$ captures *when* that probability changes most significantly. It can be empirically reconstructed by measuring how the population of |k〉 varies across many trials, each measured at a different time, and taking the magnitude of its time derivative.

We now describe the experimental procedure for reconstructing the TF distribution in a discrete quantum system with eigenstates |1〉,…,|n〉 and an observable $\hat{A}=\sum_{j=1}^{n}a_{j}|j\rangle \langle j|$.

1. **Prepare initial state.** Initialize the system in a fixed quantum state ${\hat{\rho }}_{0}$ and let it evolve unitarily under a given dynamics (open or closed systems).
2. **Perform projective measurement at time $t_{j}$.** At a chosen time $t_{j}$, perform a projective measurement of the observable ${\hat{M}}_{k}=|k\rangle \langle k|$ to check whether the system is in the target state |k〉.
3. **Repeat to obtain statistics.** Repeat the measurement independently over many trials (typically N≫1) to estimate the empirical frequency
  $$f_{k}\left(t_{j}\right)=\frac{N_{k}\left(t_{j}\right)}{N},$$
  where $N_{k}\left(t_{j}\right)$ is the number of detections in state |k〉 at time $t_{j}$. In the large-*N* limit, this converges to the probability $p_{k}\left(t_{j}\right)=\mathrm{Tr}\left(\rho_{t_{j}}{\hat{M}}_{k}\right)=\langle k|\rho_{t_{j}}|k\rangle .$
4. **Sample over a time grid.** Repeat the above procedure for a discrete set of times $\left\{t_{1},t_{2},\ldots ,t_{M}\right\}$ to obtain a time-resolved profile of the population $p_{k}\left(t\right)$. Importantly, each measurement is performed on an independent trial and only once per trajectory. This discrete-time sampling avoids continuous monitoring and thereby prevents Zeno-like inhibition of the dynamics, making it experimentally feasible in discrete systems such as qubit platforms.
5. **Estimate rate of change.** Compute the finite differences
  $$|\Delta f_{k}\left(t_{j}\right)\left|=\right|f_{k}\left(t_{j+1}\right)-f_{k}\left(t_{j}\right)|,$$
  and define the empirical rate
  $${\tilde{f}}_{k}\left(t_{j}\right)=\frac{|\Delta f_{k}\left(t_{j}\right)|}{\delta t},$$
  and define the normalized distribution
  $${\hat{\pi }}_{k}\left(t_{j}\right)=N\cdot \frac{|\Delta f_{k}\left(t_{j}\right)|}{\delta t},$$
  where $\delta t=t_{j+1}-t_{j}$ is the sampling interval and N is a normalization constant chosen so that the sum over all time bins satisfies
  $$\sum_{j=1}^{M-1}{\tilde{f}}_{k}\left(t_{j}\right)\cdot \delta t=1.$$
  This ensures that ${\tilde{f}}_{k}\left(t_{j}\right)$ can be interpreted as a properly normalized discrete approximation to the TF probability distribution.

This empirical distribution can then be used to compute statistical moments such as the mean $\mu =\sum_{j=1}^{M}t_{j}\cdot \tilde{f_{k}}\left(t_{j}\right)$, and any other higher-order momenta as $\mu^{\left(p\right)}=\sum_{j=1}^{M}t_{j}^{p}\cdot \tilde{f_{k}}\left(t_{j}\right)$, as well as variance in the time of flow. To construct the continuous-time TF probability distribution from the discrete estimate ${\hat{\pi }}_{k}\left(t_{j}\right)$, we take the joint limit where both the number of time steps *M* and the number of measurement repetitions *N* tend to infinity:

(1) $${\hat{\pi }}_{k}\left(t_{j}\right)\underset{M,N\to \infty }{⟶}N\left|\frac{d}{dt}p_{k}\left(t\right)\right|\equiv \pi_{k}\left(t\right)$$

where N is a normalization factor and $\pi_{k}\left(t\right)$ is the exact continuous-time distribution.

This analysis suggests that to obtain an analytical exact expression for the TF distribution and predict the experimental data obtained from the experiment described above, one simply needs to calculate the time derivative of the probability for the system to be detected in the state |k〉

(2) $$\pi_{k}\left(t\right)=N\left|\frac{d}{dt}p_{k}\left(t\right)\right|=N\left|\frac{d}{dt}\mathrm{Tr}\left({\hat{\rho }}_{t}{\hat{M}}_{k}\right)\right|,$$

where N is the normalization factor (over time). If $p_{k}\left(t\right)$ is monotonic, its derivative can be interpreted as a time-of-arrival (TOA) distribution when increasing, or a time-of-departure (TOD) distribution when decreasing. For non-monotonic $p_{k}\left(t\right)$, we split the time domain into increasing (TOA) and decreasing (TOD) regions, defining

(3) $$\begin{array}{cc} \pi_{k}^{\mathrm{TOA}}\left(t\right) & =N_{A}\frac{d}{dt}p_{k}\left(t\right),\;\;\;\;\mathrm{for}\;\frac{d}{dt}p_{k}\left(t\right)>0, \end{array}$$

(4) $$\begin{array}{cc} \pi_{k}^{\mathrm{TOD}}\left(t\right) & =N_{D}\left|\frac{d}{dt}p_{k}\left(t\right)\right|,\;\mathrm{for}\;\frac{d}{dt}p_{k}\left(t\right)<0, \end{array}$$

with normalization constants $N_{A}$ and $N_{D}$ over the respective time regions. Note that for the non-monotonous case, the TF distribution given by Equation (2) captures the distribution of times where the population in the state |k〉 changes, without distinguishing between arrival and departure. Note that the interpretation of Equations (2) and (3) as normalized quantum flux is analogous to the time-of-arrival (TOA) distribution in continuous systems (see [23,24]). This connection is further developed in the companion paper [25], where we introduce a general concept of the time-of-flow (TF) distribution applicable to both continuous and discrete spectra, and show that the TF distribution coincides with the TOA distribution for a particle propagating in space.

By using the master equation $\frac{d}{dt}{\hat{\rho }}_{t}=L\left({\hat{\rho }}_{t}\right)$, where L(·) is the Liouvillian superoperator [26,27], we can rewrite Equation (2) in a different form

(5) $$\pi_{k}\left(t\right)=N\left|\mathrm{Tr}\left(L\left({\hat{\rho }}_{t}\right){\hat{M}}_{k}\right)\right|=N\left|\langle k|L({\hat{\rho }}_{t})|k\rangle \right|.$$

For closed systems, we have $L\left({\hat{\rho }}_{t}\right)=-\frac{i}{\hbar }\left[\hat{H},{\hat{\rho }}_{t}\right]$, where $\hat{H}$ is the Hamiltonian of the system, and we obtain the following interesting alternative expression for the TOA distribution

(6) $$\pi_{k}\left(t\right)=N\left|\mathrm{Tr}\left({\hat{\rho }}_{t}\;{\hat{\Gamma }}_{k}\right)\right|=N\left|\frac{1}{\hbar }\mathrm{Tr}\left({\hat{\rho }}_{t}\left[\hat{H},{\hat{M}}_{k}\right]\right)\right|,$$

where $\mathrm{Tr}\left({\hat{\rho }}_{t}\;{\hat{\Gamma }}_{k}\right)=\langle \psi_{t}|{\hat{\Gamma }}_{k}|\psi_{t}\rangle$ is the quantum expectation value of the operator ${\hat{\Gamma }}_{k}\equiv -\frac{i}{\hbar }\left[\hat{H},{\hat{M}}_{k}\right]$ is the analog of the current operator for continuous systems [25]. Hence, if one designs another protocol to measure the mean value of the operator ${\hat{\Gamma }}_{k}$ at different times $t_{1},t_{2},\cdots ,t_{M}$, then we can reconstruct the TF distribution of the system. This protocol might offer a more direct measurement of the TF distribution for discrete systems such as two-level spin systems, as we will see later.

## 3. The Two-Level Spin Transition Model

### 3.1. Theoretical Results

We first consider a two-level spin transition model

(7) $$\hat{H}\left(t\right)=\frac{\hbar \omega (t)}{2}{\hat{\sigma }}_{x}$$

where ω(t) is a continuous function of t≥0. The unitary operator $\hat{U}\left(t\right)=e^{-\frac{i}{\hbar }\int_{0}^{t}\hat{H}\left(t^{\prime }\right)dt^{\prime }}=e^{-i\frac{\Omega (t)}{2}{\hat{\sigma }}_{x}}=\cos\left(\frac{\Omega (t)}{2}\right)-i\sin\left(\frac{\Omega (t)}{2}\right){\hat{\sigma }}_{x}$, where $\Omega \left(t\right)=\int_{0}^{t}\omega \left(t^{\prime }\right)dt^{\prime }$, see Appendix A, after Equation (A4), for details of the derivation. Assuming an initial state |0〉, the solution to the solution to the Schrödinger equation reads $|\psi_{t}\rangle ={\hat{U}}_{t}|0\rangle =\cos\left(\frac{\Omega (t)}{2}\right)|0\rangle -i\sin\left(\frac{\Omega (t)}{2}\right)|1\rangle$, leading to $p_{1}\left(t\right)\equiv {\left|\langle 1|\psi_{t}\rangle \right|}^{2}=\sin^{2}\left(\frac{\Omega (t)}{2}\right)$. Hence, from Equation (2) the TF distribution from state |0〉 to state |1〉 reads

(8) $$\pi_{1}\left(t\right)=N\left|\omega \left(t\right)\sin\left(\Omega (t)\right)\right|.$$

As we mentioned before, this distribution represents the TOA distribution (resp. the TOD distribution) if the derivative of $p_{1}\left(t\right)$ is positive (resp. negative). Note that we can also use the Equation (6) to find the expression of the TF distribution considering a general initial state $|\psi_{0}\rangle$

(9) $$\pi_{1}\left(t\right)=N\left|\omega \left(t\right)\cdot \langle \psi_{t}|{\hat{\sigma }}_{y}|\psi_{t}\rangle \right|=N\left|\omega \left(t\right)\cdot \langle \psi_{0}|{\hat{U}}_{t}^{†}{\hat{\sigma }}_{y}{\hat{U}}_{t}|\psi_{0}\rangle \right|,$$

where we used the commutator relation $\left[{\hat{\sigma }}_{x},|1\rangle \langle 1|\right]=i{\hat{\sigma }}_{y}$ and where the second equality was obtained from the Heisenberg representation ${\hat{\rho }}_{t}={\hat{U}}_{t}{\hat{\rho }}_{0}{\hat{U}}_{t}^{†}$. The first equality in (9) implies that the measurement of the mean value of the operator ${\hat{\sigma }}_{y}$ for t≥0 multiplied by the factor ω(t)/2 and normalized provides us an estimate of the TF distribution. The second expression in (9) gives another way to calculate explicitly the TF distribution without taking the derivative of $p_{1}\left(t\right)$. To illustrate the TF distribution in a solvable setting, we consider a two-level system with Hamiltonian $\hat{H}\left(t\right)=\frac{\hbar \omega_{0}}{2}{\hat{\sigma }}_{x}$ and initial state $|\psi_{0}\rangle =\cos\left(\frac{\theta }{2}\right)|0\rangle +\sin\left(\frac{\theta }{2}\right)|1\rangle .$ For θ≠π/2, the system evolves nontrivially toward |1〉, and the TOA distribution reads $\pi_{1}\left(t\right)=\frac{\omega_{0}}{2}\sin\left(\omega_{0}t\right),\;t\leq t_{f}=\frac{\pi }{\omega_{0}}.$ This yields a mean arrival time $\langle T_{1}\rangle =t_{f}/2$ and standard deviation $\Delta T_{1}=\frac{t_{f}}{2}\sqrt{1-\frac{8}{\pi^{2}}}$.

**Figure 1:**
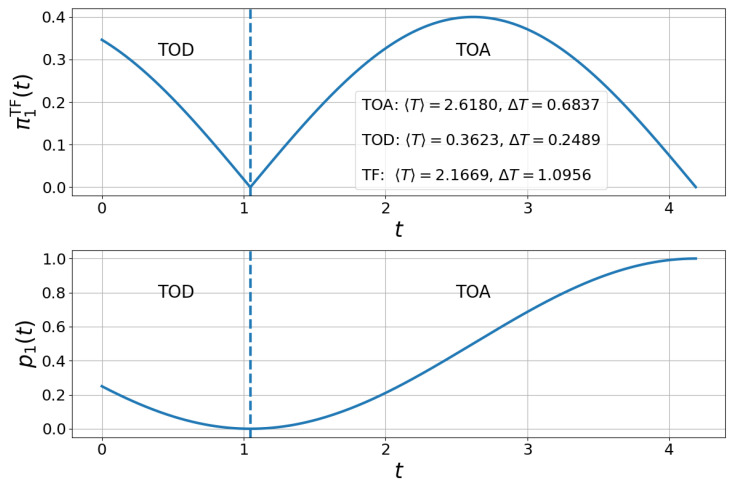
**Visual representation of TOA and TOD in an oscillating flow.** We plot the TF distribution π(t) (**top**) and probability of occupation in the target state $p_{1}\left(t\right)$ (**bottom**) for a driven two-level system with constant driving frequency and fixed phases θ=π/3 and ϕ=π/2 in (10), and we fixed $\omega_{0}=1$. The vertical dashed line at t≈1 separates the time-of-departure (TOD) regime from the time-of-arrival (TOA) regime, corresponding to the change of sign in $dp_{1}/dt$.

We can show that for a more general initial state

(10) $$|\psi_{0}\rangle =\cos\left(\frac{\theta }{2}\right)|0\rangle +e^{i\phi }\sin\left(\frac{\theta }{2}\right)|1\rangle ,$$

where θ∈[0,π], ϕ∈[0,2π), and ω(t) is a smooth real-valued function, the TF distribution reads

(11) $$\pi_{1}\left(t\right)=N\cdot \omega \left(t\right)\left[\cos(\theta )\sin(\Omega (t))-\sin(\theta )\cos(\Omega (t))\sin(\phi )\right],$$

see details of the derivation in Appendix A. Interestingly, when θ=π/3 and ϕ=π/2, see Figure 1, we see a transition between two phases: the TOD phase where the population in the target state decreases and the TOA phase where the population of the target state increases up to its maximal value.

### 3.2. Numerical Optimization

**Figure 2:**
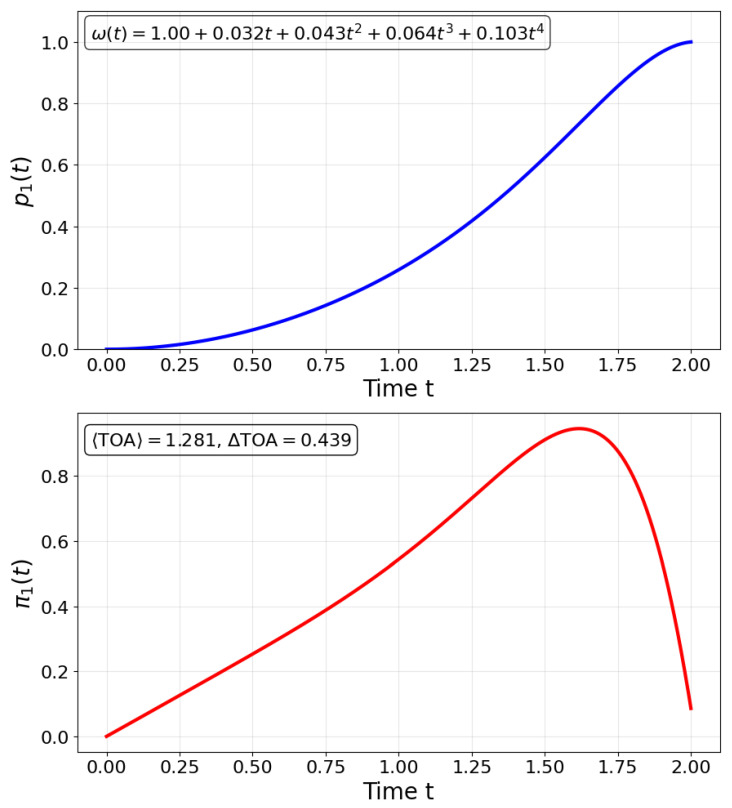
**Time-dependent control of a two-level quantum system using a polynomial protocol ω(t).** We optimize the frequency $\omega \left(t\right)=\omega_{0}+\sum_{p=1}^{4}a_{p}t^{p}$ to maximize population transfer from |0〉 to |1〉 at time *T*, while enforcing monotonic growth of $p_{1}\left(t\right)$ and regularity of the control. The cost function penalizes non-monotonic behavior and large coefficients. The optimal protocol yields a smooth population trajectory $p_{1}\left(t\right)$ and the corresponding time-of-flow (TF) distribution $\pi_{1}\left(t\right)$, enabling precise shaping of detection statistics through analytically tractable control functions.

In Figure 2, we optimize a time-dependent control protocol ω(t) driving a two-level quantum system governed by the Hamiltonian (7). Starting from the initial state |0〉, our goal is to deterministically reach the target state |1〉 at time *T*, while ensuring that the transition probability $p_{1}\left(t\right)$ increases monotonically over time. We construct ω(t) as a fourth-order polynomial $\omega \left(t\right)=\omega_{0}+\sum_{p=1}^{4}a_{p}\;t^{p}$ and define a cost function

$$J\left[a_{1},a_{2},a_{3},a_{4}\right]={\left(p_{1}\left(T\right)-1\right)}^{2}+\lambda_{\mathrm{mono}}\cdot N_{\mathrm{false}}+\lambda_{\mathrm{reg}}\sum_{i=1}^{4}a_{i}^{2},$$

where $p_{1}\left(T\right)$ is the final population of the target state, $N_{\mathrm{false}}$ counts the number of time points where $dp_{1}/dt\leq 0$, and the last term regularizes the control coefficients. The parameters $\lambda_{\mathrm{mono}}$ and $\lambda_{\mathrm{reg}}$ are penalty weights for monotonicity and regularity, respectively. The optimization exploits the exact analytical solution of the time-evolved state and provides both the population dynamics and the corresponding TF-distribution $\pi_{1}\left(t\right)$, enabling direct control over temporal detection statistics. This framework offers a versatile and computationally efficient approach to shaping quantum arrival dynamics using smooth, experimentally feasible controls.

### 3.3. The Two-Level Delta-Pulse Model: A Limiting Case

To verify the consistency of our approach, we consider the idealized delta-pulse model

(12) $$\hat{H}\left(t\right)=\frac{\pi \hbar }{2}{\hat{\sigma }}_{x}\delta \left(t-t_{0}\right),$$

describing an instantaneous transition from |0〉 to |1〉 at time $t_{0}$. This model can be obtained from Equations (7) and (8) with $\omega \left(t\right)=\pi \cdot e^{-{\left(t-t_{0}\right)}^{2}/2\sigma^{2}}/\sqrt{2\pi \sigma^{2}}$ in the limit $\lim_{\sigma \to 0}\omega \left(t\right)=\pi \delta \left(t-t_{0}\right)$. In this case, the population becomes $p_{1}\left(t\right)=\theta \left(t-t_{0}\right)$, leading to the TOA distribution

$$\pi_{1}\left(t\right)=\delta \left(t-t_{0}\right),$$

and mean TOA $\langle T_{1}\rangle =t_{0}$, as expected. The results obtained in this idealized, classical-like limit support the use of the terminology “time of arrival”. Indeed, satisfying this consistency condition, i.e., recovering a Dirac delta distribution centered at the switching time, is a minimal requirement for any approach claiming to define a TOA distribution. Other definitions should be able to reproduce this limit to justify the use of such terminology. Further delta-pulse models are analyzed in Appendix B.

## 4. Optimization of Shortcut to Adiabaticity (STA) Parameters Using TOA Distributions

### STA Model and TOA Distribution

**Figure 3:**
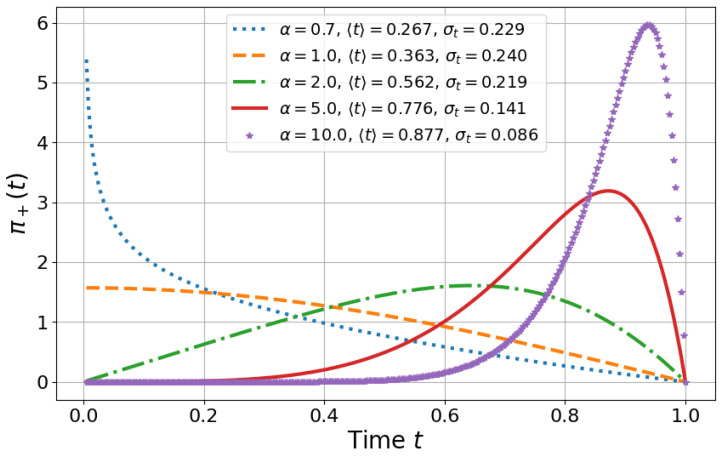
**Normalized TOA distribution for different α values.** In this figure, we plot the TOA distribution (15) for different values of α and the respective mean/standard deviation TOA (see legend within the panel) for T=1.

Let us consider a generalized spin-STA Hamiltonian [13,28]

(13) $${\hat{H}}_{\mathrm{STA}}\left(t\right)=\frac{\hbar }{2}\left[-\omega_{0}\left(\sin\left(\theta (t)\right){\hat{\sigma }}_{x}+\cos\left(\theta (t)\right){\hat{\sigma }}_{z}\right)+\dot{\theta }\left(t\right){\hat{\sigma }}_{y}\right],$$

with a flexible angle parameterization

(14) $$\theta \left(t\right)=\frac{\pi }{2}{\left(\frac{t}{T}\right)}^{\alpha },\;\alpha \geq 0$$

so that the target state is $|+\rangle =\frac{1}{\sqrt{2}}\left(|0\rangle +|1\rangle \right)$. By construction of the Hamiltonian, we know the exact solution to the time-dependent Schrödinger equation $|\psi_{t}\rangle =e^{i\phi_{t}}|n_{t}\rangle$, where the Berry phase is $\phi_{t}=\frac{\omega_{0}}{2}\int_{0}^{t}\;dt^{\prime }\sqrt{1+\frac{\dot{\theta }{\left(t^{\prime }\right)}^{2}}{\omega_{0}^{2}}}$ and where $|n_{t}\rangle =\cos\left(\frac{\theta (t)}{2}\right)|0\rangle +\sin\left(\frac{\theta (t)}{2}\right)|1\rangle$ is the solution to the stationary Schrödinger equation $H_{0}\left(t\right)|n(t)\rangle =E_{0}\left(t\right)|n(t)\rangle$, with $E_{0}\left(t\right)=\mp \frac{\hbar \omega_{0}}{2}$. From the overlap between the current state $|\psi_{t}\rangle$ and the target state |+〉, we find the probability of occupation of the target state to be $p_{+}\left(t\right)\equiv {\left|\langle +|\psi_{t}\rangle \right|}^{2}=\cos^{2}\left(\frac{\theta (t)}{2}-\frac{\pi }{4}\right)$, leading to the following expression for the normalized TF distribution

(15) $$\pi_{+}\left(t\right)=\frac{\pi \alpha }{2T}{\left(\frac{t}{T}\right)}^{\alpha -1}\cos\left(\frac{\pi }{2}{\left(\frac{t}{T}\right)}^{\alpha }\right),\;0\leq t\leq T\;,$$

which can be regarded as a TOA distribution since the analysis is restricted to a time interval where the occupation probability increases monotonically. For the linear model where α=1 (see dashed line in Figure 3) that is commonly used experimentally [13], we obtain the exact expression $\langle T_{+}\rangle =T\left(1-\frac{2}{\pi }\right)\approx 0.363\;T$ which shows a discrepancy with the target STA time *T* as well as an important spread given by $\Delta T_{+}=T\sqrt{\frac{4}{\pi }-\frac{12}{\pi^{2}}}\approx 0.240\;T.$

In Figure 3, we show that different α values lead to distinct scenarios: (i) mean TOA close to the target time *T* with relatively small standard deviation for α=5 and α=10; (ii) mean TOA closer to t=0 with relatively large standard deviation for α=0.7; and (iii) intermediate cases for α=1 and α=2. In this context, the optimal scenario is obtained for α=10, as it minimizes both the uncertainty and the difference between the target time *T* and the mean TOA, and is therefore more reliable. However, as the standard deviation of the TOA distribution decreases, the system becomes more non-adiabatic, which can introduce perturbations and noise. As a tradeoff, it may be preferable to choose a lower value of α, even at the cost of a larger standard deviation in the TOA distribution to make it smoother.

## 5. Three-Level Λ Model with Time-Dependent Detuning and Temporal Magnifier

**Figure 4:**
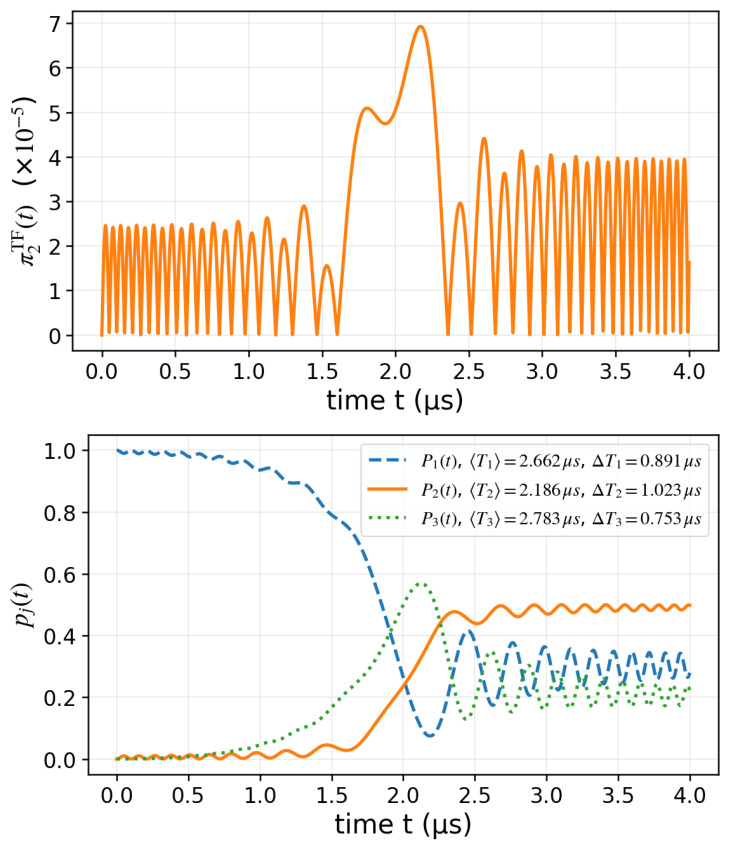
**Three-level Λ model with linear detuning.** In the bottom panel, we represent the time evolution of the populations $p_{1}\left(t\right),\;p_{2}\left(t\right),\;p_{3}\left(t\right)$ for a linear detuning Δ(t)/2π=−10→+10 MHz with constant couplings $\Omega_{1}=\Omega_{2}=1$ MHz. The legend includes TF statistics $\langle T_{j}\rangle$ and $\Delta T_{j}$ for each state, illustrating how the TF framework quantifies the characteristic durations of state occupations during the non-adiabatic passage. In the top panel, we show the normalized TF distribution $\pi_{2}^{\mathrm{TF}}\left(t\right)$ (state 2) is obtained from the population flux $dp_{2}\left(t\right)/dt$.

We consider a Λ system with ground(-like) states {|1〉,|3〉} and an excited state |2〉. The fixed Rabi couplings are $\Omega_{1}$ on the |1〉↔|2〉 transition and $\Omega_{2}$ on the |3〉↔|2〉 transition. In the rotating frame and within the rotating-wave approximation, the Hamiltonian reads

(16) $$H\left(t\right)=\left(\begin{array}{ccc} 0 & \frac{\hbar \Omega_{1}}{2} & 0\\ \frac{\hbar \Omega_{1}}{2} & \hbar \Delta (t) & \frac{\hbar \Omega_{2}}{2}\\ 0 & \frac{\hbar \Omega_{2}}{2} & 0 \end{array}\right),$$

where Δ(t) is the time-dependent single-photon detuning of the optical field driving |2〉. With fixed Rabi couplings $\Omega_{1,2}$ and initial state |1〉, we sweep the detuning once across resonance, $\Delta_{i}<0<\Delta_{f}$, implementing the standard Λ-scheme adiabatic passage [29,30]. We model the detuning as a linear ramp

(17) $$\Delta_{\mathrm{lin}}\left(t\right)=\Delta_{i}+\frac{\Delta_{f}-\Delta_{i}}{T}\;t\;,$$

with total duration *T*. To ensure adiabatic following near the avoided crossing (typically around Δ=0), the sweep rate is chosen to satisfy a Landau–Zener-type adiabaticity condition $|\dot{\Delta }|\ll \Omega_{\mathrm{eff}}^{2}$ (as the non-adiabatic transition probability is approximated by the Landau–Zener formula $P_{\mathrm{LZ}}=\exp\left(-\pi \Omega_{\mathrm{eff}}^{2}/2\left|\dot{\Delta }\right|\right)$), where $|\dot{\Delta }|=\left(\Delta_{f}-\Delta_{i}\right)/T$ and $\Omega_{\mathrm{eff}}=\sqrt{\Omega_{1}^{2}+\Omega_{2}^{2}}$ is the effective coupling between the excited state |2〉 and the bright state $|B\rangle =\left(\Omega_{1}|3\rangle +\Omega_{2}|1\rangle \right)/\Omega_{\mathrm{eff}}$. In this regime, the intermediate-state population $p_{2}\left(t\right)$ grows nearly monotonically, peaking within a narrow time window centered on the avoided crossing. This produces a single-lobed TF distribution $\pi_{2}\left(t\right)=N\;\left|\frac{d}{dt}p_{2}\left(t\right)\right|,$ localized around the crossing point. However, small-amplitude oscillations in $p_{2}\left(t\right)$ lead to significant relative changes in population, which are captured as fine structure in the TF distribution (see Figure 4). Therefore, the TF distribution acts as a temporal magnifier, revealing subtle non-adiabatic effects or internal dynamics that are otherwise hidden in population curves. While directly reconstructing $\pi_{2}\left(t\right)$ from the derivative of $p_{2}\left(t\right)$ is experimentally challenging, particularly when oscillations make the derivative noisy, we can instead employ the alternative protocol described after Equation (6). Computing the current-like operator $\hat{\Gamma }$, one finds

(18) $$\hat{\Gamma }=-\frac{\Omega_{\mathrm{eff}}}{2}\;{\hat{\sigma }}_{y}^{\left(B,2\right)}\equiv -\frac{\Omega_{\mathrm{eff}}}{2}\left(-i|B\rangle \langle 2|+i|2\rangle \langle B|\right)\;,$$

see Appendix C for details. Thus, measuring the expectation value $\langle \hat{\Gamma }\rangle$ at different times provides a direct way to reconstruct $\pi_{2}\left(t\right)$, effectively magnifying small non-adiabatic features in the dynamics.

In Figure 4, we consider two constant couplings $\Omega_{1}=\Omega_{2}=2\pi \times 1$ MHz and a linearly swept detuning Δ(t) from −10 to +10 MHz over 4 μs. Starting from state |1〉, the instantaneous dynamics is solved from the time-dependent Schrödinger equation, yielding population transfer between the three states. The plot on the bottom panel in Figure 4 displays the population dynamics of all three states, $P_{1}\left(t\right),P_{2}\left(t\right),P_{3}\left(t\right)$, with the corresponding TF mean and standard deviations included in the legend obtained from the respective TF distribution $\pi_{j}^{\mathrm{TF}}\left(t\right),\;j=1,2,3$. This illustrates how the TF protocol provides complementary timing information across the whole three-level manifold, beyond what can be inferred from instantaneous populations alone. The plot on the top panel in Figure 4 reports the normalized distribution $\pi_{2}^{\mathrm{TF}}\left(t\right)$, showing how the population of state 2 is both gained and lost during the transfer. The fast, high-amplitude oscillations observed in this figure also demonstrate that the transition is rapid and non-adiabatic mentioned above.

This application showcases the TF distribution’s utility in investigating non-trivial dynamics and opens future avenues to numerically explore protocols that could minimize such oscillations while maximizing population transfer. One suggestion we make is the use of physics-informed neural networks for this task [31].

## 6. Time-of-Flow Distribution and Decoherence in Open Systems

### 6.1. Time-of-Flow Distribution for a General Markovian System

Consider the Markovian master equation

(19) $$\frac{d}{dt}{\hat{\rho }}_{t}=L\left({\hat{\rho }}_{t}\right)=-\frac{i}{\hbar }\left[\hat{H},{\hat{\rho }}_{t}\right]-\sum_{j}\frac{\gamma_{j}}{2}\left[{\hat{L}}_{j},\left[{\hat{L}}_{j},{\hat{\rho }}_{t}\right]\right],$$

where ${\hat{\rho }}_{t}$ is the density matrix, $\hat{H}$ is a time-independent Hamiltonian, ${\hat{L}}_{j}$ are time-independent Lindblad operators, and $\gamma_{k}$ are the coupling constants.

We recall that the TOA distribution at the fixed state |k〉 is given by

(20) $$\pi_{k}\left(t\right)=\left|\frac{d}{dt}\mathrm{Tr}\left({\hat{\rho }}_{t}{\hat{M}}_{k}\right)\right|,$$

where ${\hat{M}}_{k}=|k\rangle \langle k|$ is the projector onto the state |k〉.

From the master Equation (19), we find that

$$\begin{array}{cc} \frac{d}{dt}\mathrm{Tr}\left({\hat{\rho }}_{t}{\hat{M}}_{k}\right) & =-\frac{i}{\hbar }\mathrm{Tr}\left({\hat{\rho }}_{t}\left[-\hat{H},\hat{M}\right]\right)+\mathrm{Tr}\left({\hat{\rho }}_{t}D\left({\hat{M}}_{k}\right)\right)\\ \; & =\mathrm{Tr}(\rho_{t}L^{†}\left({\hat{M}}_{k}\right)), \end{array}$$

where the dissipator $D\left({\hat{M}}_{k}\right)=-\sum_{j}\frac{\gamma_{j}}{2}\left[{\hat{L}}_{j},\left[{\hat{L}}_{j},{\hat{M}}_{k}\right]\right]$ and $L^{†}\left(\cdot \right)$ is the Hermitian conjugate of the Liouville super-operator. Hence, we find that

(21) $$\pi_{k}\left(t\right)=N\left|\mathrm{Tr}\left({\hat{\rho }}_{t}L^{†}\left({\hat{M}}_{k}\right)\right)\right|\;,$$

where N is the normalization factor. Notice that for a closed quantum system, we find the Formula (6), as expected. Also, the operator $\hat{\Gamma }\equiv L^{†}\left({\hat{M}}_{k}\right)$ can be viewed as the analog of a current operator, as in Equation (6) and provides a more direct way to access the TF distribution, provided that $\hat{\Gamma }$ is experimentally accessible. An example will be discussed in Section 6.5.

### 6.2. Quantum Speed Limit Associated to the TF Distribution

From the master Equation (19), we find that

$$\begin{array}{cc} \frac{d}{dt}p_{k}\left(t\right) & =\mathrm{Tr}({\hat{\rho }}_{t}L^{†}\left({\hat{M}}_{k}\right)), \end{array}$$

Hence, we find that

(22) $$\left|\frac{d}{dt}p_{k}\left(t\right)\right|=|\mathrm{Tr}\left({\hat{\rho }}_{t}L^{†}\left({\hat{M}}_{k}\right)\right)\left|\leq \right|\sqrt{\mathrm{Tr}(L^{†}{\left({\hat{M}}_{k}\right)}^{2})|}\;,$$

where we used the Cauchy–Schwarz inequality.

Integrating over time from 0 to τ leads to

$$|p_{k}\left(t\right)-p_{k}\left(0\right)|\leq \int_{0}^{T}\left|\frac{d}{dt}p_{k}\left(t\right)\right|\leq T\sqrt{\mathrm{Tr}(L^{†}{\left({\hat{M}}_{k}\right)}^{2})|}$$

After parameterizing $p_{k}\left(T\right)-p_{k}\left(0\right)=\theta_{T}-\theta_{0}\equiv \delta \theta$, we find

(23) $$T\geq \tau_{\mathrm{TF}}\equiv \frac{\delta \theta }{\sqrt{\left|\mathrm{Tr}(L^{†}{\left({\hat{M}}_{k}\right)}^{2})\right|}}.$$

This time-of-flow-like QSL or TF-QSL for transition is the analog to MT-like QSL for open quantum systems, see [20]. Note that for closed system, we obtain the following TF-QSL

(24) $$\tau_{\mathrm{TF}}=\frac{\hbar \;\delta \theta }{2\Delta_{k}H}\;,$$

where $\Delta_{k}H\equiv \sqrt{\mathrm{Tr}\left({\hat{H}}^{2}\;{\hat{M}}_{k}\right)-\mathrm{Tr}{\left(\hat{H}\;{\hat{M}}_{k}\right)}^{2}}=\sqrt{\langle k|{\hat{H}}^{2}|k\rangle -\langle k|\hat{H}{|k\rangle }^{2}}$ is the standard deviation of the Hamiltonian with respect to the state |k〉. To derive the bound (24) from the bound (23), we use that $|\mathrm{Tr}\left(L^{†}{\left({\hat{M}}_{k}\right)}^{2}\right))|=\left(1/\hbar \right)|\mathrm{Tr}\left({\left[\hat{H},{\hat{M}}_{k}\right]}^{2}\right)|=\left(1/\hbar \right)\left|\mathrm{Tr}\left(\hat{H}{\hat{M}}_{k}\hat{H}{\hat{M}}_{k}\right)-\mathrm{Tr}\left({\hat{H}}^{2}{\hat{M}}_{k}^{2}\right)\right|=\left(1/\hbar \right){\left(\Delta_{k}H\right)}^{2}$, as ${\hat{M}}_{k}^{2}={\hat{M}}_{k}$ and $\mathrm{Tr}\left(\hat{A}{\hat{M}}_{k}\right)=\langle k|\hat{A}|k\rangle$. This result provides a transition-specific QSL framed in terms of experimentally accessible observables via projective measurements at discrete time steps on independently prepared systems, avoiding Zeno inhibition. Unlike traditional QSLs based on fidelity or Bures angle, this bound focuses on the physical population of a target state and connects naturally with experimentally measured quantities, such as the time-of-flow distribution. It is particularly well suited for discrete quantum systems, such as qubits, where occupation probabilities are the primary observable of interest.

Other approaches related to observables, such as those based on quantum speed limits (QSL) for observables [32,33], have been previously explored; however, these typically emphasize “what you observe" by bounding the rate of change of the expectation value of a Hermitian operator *A*, using its variance with respect to the quantum state. In contrast, the time-of-flow (TF) approach addresses the complementary question of “when you observe" by focusing on the dynamics of the projective measurement operator $M_{k}$, independent of the state. The resulting distribution $\pi_{k}\left(t\right)=N\left|{\dot{p}}_{k}\left(t\right)\right|$ characterizes the time statistics of detection events, from which we derive an uncertainty relation and a quantum speed limit based on the energy variance *with respect to the measurement operator*. While the mathematical tools (e.g., norm inequalities) may be similar, the TF-QSL differs both formally and conceptually from observable-based QSLs by grounding the bound in a probabilistic and operational measurement framework.

### 6.3. Lower Bound for the Standard Deviation of the TF Distribution and Uncertainty Relation

By Chebyshev’s inequality, for any random variable T with distribution π(t) and finite variance ${\left(\Delta T\right)}^{2}$, we find the following lower bound:

(25) $$\Delta T\geq \frac{1}{3\sqrt{3}}\;\frac{1}{\pi_{\max}},$$

see [25] for details of the derivation. This inequality shows that the standard deviation Δt of the TF distribution cannot be made arbitrarily small if $\pi_{\max}$ is bounded, enforcing a minimal temporal spread inversely proportional to the peak height of the TF distribution. It provides a time-uncertainty relation that complements the mean time analysis.

Now, combining the TF-QSL (23) with the lower bound of the standard deviation (25), we find

(26) $$\Delta T\geq \frac{1}{3\sqrt{3}}\;\frac{\delta \theta }{\sqrt{\left|\mathrm{Tr}(L^{†}{\left({\hat{M}}_{k}\right)}^{2})\right|}}=\frac{1}{3\sqrt{3}}\cdot \tau_{\mathrm{TF}},$$

where δθ=p(T)−p(0). To find this result, it suffices to note that $\pi_{\max}\leq N\sqrt{\left|\mathrm{Tr}(L^{†}{\left({\hat{M}}_{k}\right)}^{2})\right|}$, as previously shown, and that $N^{-1}=\int_{0}^{T}\left|\frac{dp_{k}\left(t\right)}{dt}\right|\;dt\geq \left|\int_{0}^{T}\frac{dp_{k}\left(t\right)}{dt}\;dt\right|=p_{k}\left(T\right)-p_{k}\left(0\right)$.

Note that using the TF-QSL for closed systems given in Equation (24), we find the following uncertainty relation

(27) $$\Delta T\cdot \Delta_{k}H\geq \eta \cdot \hbar ,$$

where $\eta =\frac{1}{6\sqrt{3}}\delta \theta =\frac{1}{6\sqrt{3}}\left|p_{k}\left(T\right)-p_{k}\left(0\right)\right|$.

The inequality (27) provides a novel and operationally grounded uncertainty relation for the TF. This bound emerges from a combination of Chebyshev’s inequality and the TF-based quantum speed limit, and offers a stochastic interpretation based on a clearly defined protocol that measures the TF probability distribution associated with the random variable T. Crucially, it links the temporal spread of the arrival distribution to both the maximal sharpness of the distribution and the energy scale of the evolution, yielding a genuine Heisenberg-like bound. This result shows that temporal resolution in quantum transitions cannot be made arbitrarily small without increasing the energetic resources or sacrificing transition amplitude. As such, it offers a new tool for assessing the performance and limits of quantum control protocols and time-resolved measurements.

Note that in a companion paper [25], an alternative time–energy uncertainty relation is derived, which depends on the standard deviation of the Hamiltonian with respect to the initial state $\rho_{0}$, defined as $\Delta H\equiv \sqrt{\mathrm{Tr}\left({\hat{H}}^{2}{\hat{\rho }}_{0}\right)-\mathrm{Tr}{\left(\hat{H}{\hat{\rho }}_{0}\right)}^{2}}$. This formulation is particularly useful for Hamiltonians with continuous spectra. The two uncertainty relations are complementary: the one presented in this article, Equation (27), is applicable to Hamiltonians with discrete spectra and provides a bound for ΔT that depends specifically on the energy dispersion within the projector state and offers a direct relation with the QSL as shown in (26), which is applicable for open quantum systems. In contrast, the state-dependent bound in [25], which extends naturally to both discrete and continuous spectra, provides complementary information on ΔT, as it is related to the energy dispersion within the state. Since it depends on the state transfer δθ, it is also measurement-dependent. Moreover, this bound is restricted to closed quantum systems, whereas the bound presented here can be naturally extended to open dynamics, as shown in Equation (26).

### 6.4. Dephasing Model and Quantum Speed Limits

We now illustrate the applicability of the TF distribution framework to open quantum systems by considering a paradigmatic pure dephasing model governed by the Lindblad master equation:

(28) $$\frac{d}{dt}{\hat{\rho }}_{t}=-\frac{\gamma }{2}\left[{\hat{\sigma }}_{z},\left[{\hat{\sigma }}_{z},{\hat{\rho }}_{t}\right]\right],$$

with initial state ${\hat{\rho }}_{0}=|+\rangle \langle +|$. The off-diagonal coherence elements in the density matrix decay exponentially, with a characteristic decoherence time $\tau_{D}={\left(\gamma \Delta {\hat{L}}_{z}^{2}\right)}^{-1}=1/\gamma$ [34]. Within our TF distribution framework, the probability of transition from |+〉 to the orthogonal state |−〉 evolves as

(29) $$p_{-}\left(t\right)=\frac{1}{2}\left(1-e^{-2\gamma t}\right),$$

yielding the normalized TF distribution (see Equation (21))

(30) $$\pi_{-}\left(t\right)=N\left|\frac{d}{dt}p_{-}\left(t\right)\right|=2\gamma e^{-2\gamma t}.$$

This distribution is properly normalized, and provides a well-defined mean arrival time

(31) $$\langle T_{-}\rangle =\Delta T_{-}=\frac{1}{2\gamma },$$

both equal to half the decoherence time $\tau_{D}$. This result demonstrates that the TF distribution captures the temporal scale of decoherence via observable transitions.

We now evaluate the quantum speed limit (QSL) associated with this transition using the TF framework. From (23) with the projection operator M=|−〉〈−| and $L^{†}\left(M\right)=\gamma \left(\sigma_{z}M\sigma_{z}-M\right)=\gamma \sigma_{x},$ we obtain

(32) $$\mathrm{Tr}\left[L^{†}{\left(M\right)}^{2}\right]=\gamma^{2}\mathrm{Tr}\left(\sigma_{x}^{2}\right)=2\gamma^{2}.$$

Since $p_{-}\left(0\right)=0$ and $p_{-}\left(\infty \right)=\frac{1}{2}$, the population change is δθ=1/2. We obtain the TF-based QSL

(33) $$\tau_{\mathrm{TF}}=\frac{1}{2\sqrt{2}\;\gamma }.$$

This result can be directly compared to the fidelity-based Mandelstam–Tamm-type bound derived by del Campo et al. [20], which yields

(34) $$\tau_{\mathrm{MT}}=\frac{1}{\sqrt{2}\;\gamma }.$$

Our TF-based QSL is smaller by a factor 1/2, which reflects the fundamentally different physical quantities they constrain: MT’s bound limits the distinguishability between initial and final states based on fidelity, while our TF-QSL captures the minimal time required to realize a detectable population transfer in a specified state via projective measurements. In contrast to global overlap-based speed limits, the TF-QSL directly links the dynamical generator L and the projection operator *M*, offering a physically transparent and experimentally accessible temporal bound for open system transitions.

In addition to bounding the mean arrival time, the TF framework also yields a fundamental lower bound on the standard deviation $\Delta T_{-}$ of the TF distribution that we can derive from Equation (27)

(35) $$\Delta T_{-}\geq \frac{1}{3\sqrt{3}}\cdot \tau_{\mathrm{TF}}=\frac{1}{6\sqrt{6}\;\gamma }.$$

This inequality reveals that the temporal spread of arrival times cannot be made arbitrarily small, even in the idealized exponential decay regime. It complements the TF-QSL by capturing a fundamental irreducible fluctuation in the arrival statistics, which is effectively a Heisenberg-type temporal uncertainty rooted in the transition probability and dynamical generator. In this example, we found $\Delta T_{-}=1/\left(2\gamma \right)$, which is about 7.35 times the limit in (35).

### 6.5. Explicit TF–QSL Bound in a Hadamard Dephasing Model

To demonstrate the operational computability of the TF–QSL bound in open dynamics, we now consider a minimal model consisting of a Hadamard-like rotation with a Markovian dephasing channel along *z* with rate γ:

(36) $$\frac{d}{dt}{\hat{\rho }}_{t}=L\left({\hat{\rho }}_{t}\right)=-\frac{i}{\hbar }\left[\hat{H},{\hat{\rho }}_{t}\right]+\frac{\gamma }{2}\left({\hat{\sigma }}_{z}{\hat{\rho }}_{t}{\hat{\sigma }}_{z}-{\hat{\rho }}_{t}\right)\;,$$

where the Hamiltonian is proportional to the Hadamard gate $\hat{h}=\left({\hat{\sigma }}_{x}+{\hat{\sigma }}_{z}\right)/\sqrt{2}$

(37) $$\hat{H}=\frac{\hbar \omega_{0}}{2}\;\frac{{\hat{\sigma }}_{x}+{\hat{\sigma }}_{z}}{\sqrt{2}}\;,$$

where the Hadamard gate $\hat{h}$ is transforming the computational basis {|0〉,|1〉} to the diagonal basis {|+〉,|−〉} from the relations $\hat{h}|0\rangle =|+\rangle$ and $\hat{h}|1\rangle =|-\rangle$. Hence, the strategy is to make a transition from the computational to the diagonal basis, thus we consider the initial state |0〉 and the target state |+〉. To realize the protocol, we then use the measurement projector $M_{+}=|+\rangle \langle +|$. The goal is to use the protocol to study the dynamics of the transition between the initial and the target states and to find a lower bound for the dispersion of the TF distribution as well as an estimate of the minimal resolution of the detector to analyze this transition. From the general TF–QSL (23) and (26), we obtain the explicit lower bound

(38) $$\Delta T_{+}\geq \frac{1}{3\sqrt{3}}\tau_{\mathrm{TF}}=\frac{1}{3\sqrt{3}}\frac{\delta \theta }{\sqrt{\frac{\omega_{0}^{2}}{4}+\frac{\gamma^{2}}{2}}},$$

where $\delta \theta =p_{+}\left(T\right)-p_{+}\left(0\right)$ is the net population transfer; see Appendix D for details of the derivation. This bound immediately shows how the coherent drive $\omega_{0}$ and the dephasing rate γ compete in setting the minimal temporal resolution of the transfer to |+〉.

**Figure 5:**
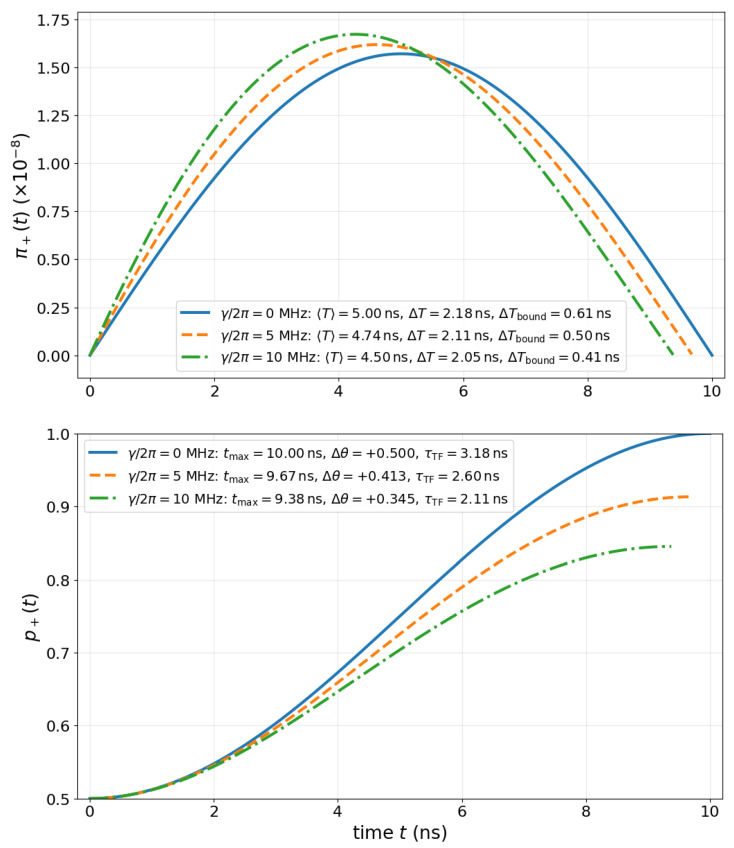
**TF Distributions and time-uncertainty bound in a Hadamard dephasing V.** Normalized TF distributions $\pi \left(t\right)=|dp_{+}\left(t\right)/dt|$ (in units of 1/ns) for increasing dephasing rates γ/2π=0,5, 10 MHz, with legend reporting 〈T〉, ΔT, and the theoretical lower bound $\Delta T_{\mathrm{bound}}=\tau_{\mathrm{TF}}/\left(3\sqrt{3}\right)$ is represented on the top panel. On the bottom panel, we show the population dynamics $p_{+}\left(t\right)=\mathrm{Tr}\left(\rho_{t}|+\rangle \langle +|\right)$ and effective angular change δθ for each case. The transition becomes slower and less complete as dephasing increases, validating the predicted scaling of the bound (38).

To illustrate the behavior of the TF distribution and its associated QSL bound across different decoherence regimes, we numerically solve the open-system dynamics defined by Equation (37). Figure 5 displays both the normalized time-of-flow (TF) distribution $\pi_{+}\left(t\right)$ and the population dynamics $p_{+}\left(t\right)=\mathrm{Tr}\left[\rho_{t}M_{+}\right]$ for three representative values of the dephasing rate γ/2π=0,5,10 MHz. For each case, we extract the TF distribution from the transition probability curve using $\pi_{+}\left(t\right)=N\left|dp_{+}\left(t\right)/dt\right|$, where N is the normalization factor, compute its mean $\langle T_{+}\rangle$ and standard deviation $\Delta T_{+}$, and compare the latter to the theoretical bound (38). The lower plot shows how increased dephasing slows down the transition and reduces the effective angular displacement $\delta \theta =p_{+}\left(T\right)-p_{+}\left(0\right)$, thereby enlarging the minimal allowed time uncertainty. This provides a concrete demonstration of the TF–QSL constraint under dissipation, with the lower bound serving as a resolution benchmark for the detection of population transfer in realistic open quantum devices.

Note that the TF distribution $\pi_{+}\left(t\right)$ can be directly obtained by measuring the expectation value of the current-like operator

(39) $$\hat{\Gamma }\equiv -\frac{\omega_{0}}{2\sqrt{2}}\;{\hat{\sigma }}_{y}-\frac{\gamma }{2}\;{\hat{\sigma }}_{x}$$

at different times, see Equation (21) and Appendix D. Normalizing this quantity over time yields the distribution $\pi_{+}\left(t\right)$, providing a simple and direct route to extracting a time distribution from experimental data.

## 7. Concluding Remarks

In this work, we introduced a general and operationally meaningful definition of the time-of-flow (TF) distribution for discrete quantum systems. The TF distribution, derived from the rate of change of population in a target state, provides a unifying and experimentally accessible framework to characterize the timing of quantum transitions. In monotonic regimes, it admits a natural interpretation as a time-of-arrival (TOA) or time-of-departure (TOD) distribution, and we verified its consistency by recovering the expected delta-function behavior in the limiting delta-pulse model.

We applied this framework to several scenarios: (i) an analytically solvable two-level transition model, including optimization of polynomial control protocols, (ii) optimization of smooth shortcut-to-adiabaticity (STA) protocols, (iii) characterization of multi-level dynamics through a three-level Λ model with time-dependent detuning, (iv) analysis of decoherence in open systems using the Lindblad equation, and (v) analysis of TF-based quantum speed limits and uncertainty relations for a Hadamard dephasing model. In each case, the TF distribution enabled both analytical insight and numerical control over the temporal statistics of state transitions.

Beyond descriptive power, the TF distribution enabled the derivation of two fundamental results: a quantum speed limit (TF-QSL) specific to population transfer, and a Heisenberg-like uncertainty relation that bounds the temporal resolution of transitions in terms of the peak height of the TF distribution and the system’s dynamical generator. These results connect temporal features of state dynamics to underlying physical resources, offering new tools to assess and optimize quantum control strategies.

Looking ahead, this framework can guide the design of robust STA protocols by minimizing the mean TOA and its uncertainty while mitigating decoherence. Cost functions combining mean time, standard deviation, and fidelity offer principled criteria for optimization. The TF distribution can also serve as a diagnostic of non-adiabatic behavior and as an observable in future experimental studies. Extensions to more multi-level systems, time-dependent noise, and machine-learning-based optimization in the context of quantum computing are natural next steps for this approach.

Looking ahead, the TF framework could guide the design of robust STA protocols by minimizing both mean transition time and its uncertainty and could serve as a diagnostic tool for non-adiabatic dynamics in near-term quantum devices. Extensions to multi-level systems, noisy intermediate-scale quantum (NISQ) architectures, and machine-learning-based optimization represent natural next steps. Experimentally, the TF distribution could be reconstructed in superconducting qubits or trapped-ion platforms, providing direct benchmarks of control protocols and new probes of temporal quantum uncertainty.

## Figures and Tables

**Figure A1:**
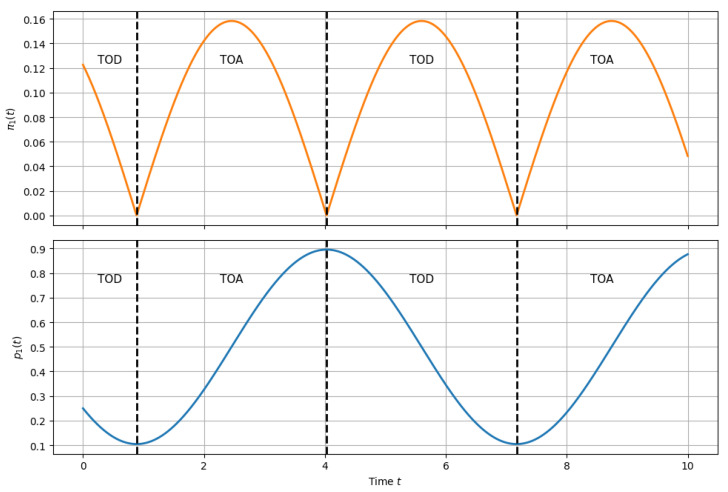
**Transition dynamics and time-of-flow distribution in a two-level system.** Plots of the state occupation probability $p_{1}\left(t\right)$ (**bottom**), the non-normalized time-of-flow (TF) distribution $\left|\frac{dp_{1}}{dt}\right|$ (**top**), and the time-of-arrival (TOA) and time-of-departure (TOD) regions for a two-level system with constant Hamiltonian $\hat{H}\left(t\right)=\frac{\hbar \omega_{0}}{2}{\hat{\sigma }}_{x}$ and initial state $|\psi_{0}\rangle =\cos\left(\theta /2\right)|0\rangle +e^{i\phi }\sin\left(\theta /2\right)|1\rangle$, with θ=π/3 and ϕ=π/4. TOA regions correspond to $\frac{dp_{1}}{dt}>0$, while TOD regions correspond to $\frac{dp_{1}}{dt}<0$. We fixed $\omega_{0}=1$.

**Figure A2:**
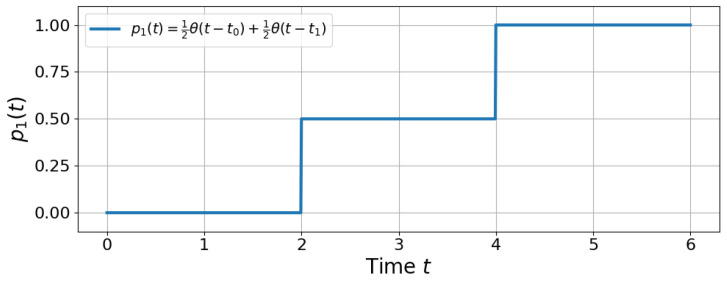
**Piecewise population function $p_{1}\left(t\right)$ showing the arrival in the state |1〉.** The flow shows two steps corresponding to two TOA regions for 0≤t<4 and 2<t≤6. As expected, the mean TOA is $\frac{1}{2}\left(t_{0}+t_{1}\right)=3$ and the standard deviation is $\frac{1}{2}\left(t_{1}-t_{0}\right)=1$.

**Figure A3:**
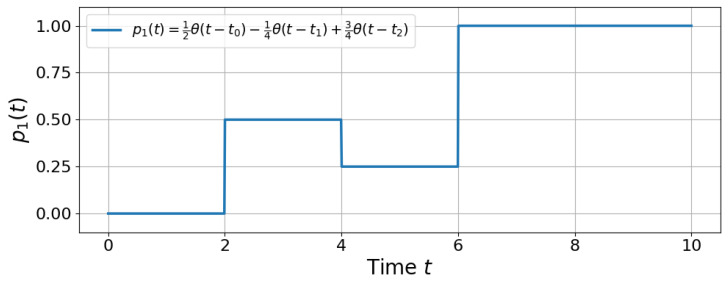
**Piecewise population function $p_{1}\left(t\right)$ with TOA and TOD.** The flow alternates between TOA regions for 0≤t<4 and 4<t≤10, and a TOD region for 2<t<6.

## Data Availability

The programming code supporting the findings of this study is openly available on GitHub at https://github.com/mathieubeau/TF_Quantum/blob/main/Codes_for_TF_discrete_paper_arxiv_org_abs_2504_09571.ipynb, accessed on 21 September 2025.

## Abbreviations

The following abbreviations are used in this manuscript:

TF	Time-of-flow
TOA	Time-of-arrival
TOD	Time-of-departure
QSL	Quantum Speed Limit
TF-QSL	TF-based quantum speed limit
MT-QSL	Mandelstam-Tamm quantum speed limit
STA	Shortcut to adiabaticity

## Appendix A. Generalization of the Formula (8)

Consider a two-level system evolving under the time-dependent Hamiltonian

(A1) $$\hat{H}\left(t\right)=\frac{\hbar \omega (t)}{2}{\hat{\sigma }}_{x},$$

with initial state

(A2) $$|\psi_{0}\rangle =\cos\left(\frac{\theta }{2}\right)|0\rangle +e^{i\phi }\sin\left(\frac{\theta }{2}\right)|1\rangle ,$$

where θ∈[0,π], ϕ∈[0,2π), and ω(t) is a smooth real-valued function.

Let $\Omega \left(t\right)=\int_{0}^{t}\omega \left(t^{\prime }\right)dt^{\prime }$. Since $\hat{H}\left(t\right)\propto {\hat{\sigma }}_{x}$, the time-evolution operator reads

(A3) $$\hat{U}\left(t\right)=\exp\left(-\frac{i}{\hbar }\int_{0}^{t}\hat{H}\left(t^{\prime }\right)dt^{\prime }\right)=\exp\left(-i\frac{\Omega (t)}{2}{\hat{\sigma }}_{x}\right).$$

First, let us prove the identity

(A4) $$e^{-i\phi \sigma_{x}}=\cos\left(\phi \right)I-i\sin\left(\phi \right)\sigma_{x}$$

using Taylor series

$$\begin{array}{cc} e^{-i\phi \sigma_{x}}\; & =\sum_{k=0}^{\infty }\frac{{\left(-i\phi \right)}^{k}}{k!}\;\sigma_{x}^{k}\\ \; & =\sum_{m=0}^{\infty }\frac{{\left(-i\phi \right)}^{2m}}{(2m)!}\;I+\sum_{m=0}^{\infty }\frac{{\left(-i\phi \right)}^{2m+1}}{(2m+1)!}\;\sigma_{x}.\\ \; & =\cos\left(\phi \right)\;I-i\sin\left(\phi \right)\;\sigma_{x} \end{array}$$

as

$$\sigma_{x}^{2m}=I,\;\sigma_{x}^{2m+1}=\sigma_{x}\;,\;m\in N.$$

and as

$$\begin{array}{cc} \sum_{m=0}^{\infty }\frac{{\left(-i\phi \right)}^{2m}}{(2m)!}\; & =\sum_{m=0}^{\infty }\frac{{\left(-1\right)}^{m}\phi^{2m}}{(2m)!}=\cos\phi ,\\ \sum_{m=0}^{\infty }\frac{{\left(-i\phi \right)}^{2m+1}}{(2m+1)!}\; & =-i\sum_{m=0}^{\infty }\frac{{\left(-1\right)}^{m}\phi^{2m+1}}{(2m+1)!}=-i\sin\phi . \end{array}$$

Now, using the identity (A4) and setting $\phi =\frac{\Omega (t)}{2}$, we find

(A5) $$\hat{U}\left(t\right)=\cos\left(\frac{\Omega (t)}{2}\right)\;I-i\sin\left(\frac{\Omega (t)}{2}\right)\;\sigma_{x}$$

From the expression of the solution to the Schrödinger equation,

$$\begin{array}{cc} |\psi (t)\rangle & =\hat{U}\left(t\right)|\psi_{0}\rangle \\ \; & =\left(\cos\left(\frac{\theta }{2}\right)\cos\left(\frac{\Omega (t)}{2}\right)-ie^{i\phi }\sin\left(\frac{\theta }{2}\right)\sin\left(\frac{\Omega (t)}{2}\right)\right)|0\rangle \\ \; & +\left(e^{i\phi }\sin\left(\frac{\theta }{2}\right)\cos\left(\frac{\Omega (t)}{2}\right)-i\cos\left(\frac{\theta }{2}\right)\sin\left(\frac{\Omega (t)}{2}\right)\right)|1\rangle \end{array}$$

we can compute the probability of finding the system in state |1〉:

(A6) $$\begin{array}{cc} p_{1}\left(t\right) & ={\left|\langle 1|\psi \left(t\right)\rangle \right|}^{2}\\ \; & =\sin^{2}\left(\frac{\theta }{2}\right)\cos^{2}\left(\frac{\Omega (t)}{2}\right)+\cos^{2}\left(\frac{\theta }{2}\right)\sin^{2}\left(\frac{\Omega (t)}{2}\right)\\ \; & \;-\frac{1}{2}\sin\left(\theta \right)\sin\left(\Omega \left(t\right)\right)\sin\left(\phi \right). \end{array}$$

Differentiating $p_{1}\left(t\right)$ yields the TF distribution:

(A7) $$\begin{array}{cc} \frac{d}{dt}p_{1}\left(t\right) & =\frac{\omega (t)}{2}\left[\cos(\theta )\sin(\Omega (t))-\sin(\theta )\cos(\Omega (t))\sin(\phi )\right], \end{array}$$

(A8) $$\begin{array}{cc} \pi_{1}\left(t\right) & =N\left|\frac{d}{dt}p_{1}\left(t\right)\right|, \end{array}$$

where N is the normalization constant for the TF distribution.

When ϕ=0, we obtain

$$\frac{d}{dt}p_{1}\left(t\right)=\frac{\omega (t)}{2}\cos\left(\theta \right)\sin\left(\Omega \left(t\right)\right).$$

If $\omega \left(t\right)=\omega_{0}$, we find after normalization and restriction in the TOA region ($t\leq \pi /\omega_{0}$)

$$\pi_{1}\left(t\right)=\frac{\omega_{0}}{2}\sin\left(\omega_{0}t\right),$$

as shown in the main body of the paper. A particular case where θ=π/3 is shown in Figure A1.

When ϕ=π/2, we obtain

$$\frac{d}{dt}p_{1}\left(t\right)=\frac{\omega (t)}{2}\sin\left(\Omega \left(t\right)-\theta \right).$$

So, if $\omega \left(t\right)=\omega_{0}$, we find that the TF distribution can be interpreted as a TOD distribution for $0\leq t\leq \theta /\omega_{0}$ and $\left(\left(2k-1\right)\pi +\theta \right)/\omega_{0}\leq t\leq \left(2k\pi +\theta \right)/\omega_{0}$, see Figure A1.

## Appendix B. Limiting Case: The Delta-Pulse Model

We can construct a similar model to fill the state |1〉 in two steps, e.g., $p_{1}\left(t\right)=\frac{1}{2}\left(\theta \left(t-t_{0}\right)+\theta \left(t-t_{1}\right)\right)$, which means that half of the spins are expected to be observed in the state |1〉 between $t_{0}$ and $t_{1}$ and the other half after $t_{1}$. Note that this model can be obtained using a pulse model with a Hadamard gate (operator $\hat{h}=\frac{1}{\sqrt{2}}\left({\hat{\sigma }}_{x}+{\hat{\sigma }}_{z}\right)$) to obtain a $|+\rangle =\frac{1}{\sqrt{2}}\left(|0\rangle +|1\rangle \right)$ state between $t_{0}$ and $t_{1}$, and then a combined Z-gate and Hadamard gate ($\hat{h}\;{\hat{\sigma }}_{z}$ operator) to obtain a $|-\rangle =\frac{1}{\sqrt{2}}\left(|0\rangle -|1\rangle \right)$ and |1〉 after applying the Z-gate and the Hadamard gate subsequently.

Using Equation (2), we now find $\pi_{1}\left(t\right)=\frac{d}{dt}p_{1}\left(t\right)=\frac{1}{2}\delta \left(t-t_{0}\right)+\frac{1}{2}\delta \left(t-t_{1}\right)$, whence $\langle T_{1}\rangle =\frac{t_{0}+t_{1}}{2}$, which can be interpreted as the TOA distribution for $\frac{d}{dt}p_{1}\left(t\right)>0$, see Figure A2. This means that we expect half of the spins to *arrive* in the state |1〉 at $t_{0}$ and the other half at $t_{1}$, and thus, the average time of arrival should be the average between these two times, as is consistent with the proposed model. Also, the standard deviation for this two-step model is non-zero, $\Delta T_{1}=\sqrt{\frac{1}{2}t_{0}^{2}+\frac{1}{2}t_{1}^{2}-{\left(\frac{1}{2}t_{0}+\frac{1}{2}t_{1}\right)}^{2}}=\frac{1}{2}\left(t_{1}-t_{0}\right)$, as expected.

We could of course generalize the approach to a multi-step model with $p_{1}\left(t\right)=\sum_{l=0}^{n}a_{l}\theta \left(t-t_{l}\right)$, where $t_{0}<t_{1}<\cdots <t_{n}$ and where $a_{l}>0,\;\forall l=1,\ldots ,n$ can be interpreted as the change of probabilities of occupation of the state |1〉 between two subsequent times $t_{l-1}$ and $t_{l}$. Here we have $a_{0}+a_{1}+\cdots +a_{n}=1$ and the probabilities of occupations at times $t_{l}$ are then $\sum_{m=0}^{l}a_{m}$. Of course, we can also construct TOD distribution by taking $a_{l}<0,\;\forall l=1,\ldots ,n$.

It is also possible to study non-monotonic cases, where the dynamics alternate between TOA and TOD phases, resulting in an overall positive or negative net flow. For example, take $p_{1}\left(t\right)=\frac{1}{2}\theta \left(t-t_{0}\right)-\frac{1}{4}\theta \left(t-t_{1}\right)+\frac{3}{4}\theta \left(t-t_{2}\right)$ with $t_{0}=2,\;t_{1}=4,\;t_{2}=6$, see Figure A3, where the flow alternates between TOA regions for 0≤t<4 and 4<t≤10, and a TOD region for 2<t<6. So, the normalized TOA distribution is

$$\pi_{1}^{\left(\mathrm{TOA}\right)}=\frac{2}{5}\delta \left(t-t_{0}\right)+\frac{3}{5}\delta \left(t-t_{2}\right)$$

and the normalized TOD distribution is

$$\pi_{1}^{\left(\mathrm{TOD}\right)}=\delta \left(t-t_{1}\right).$$

Hence, the mean TOA is $\langle T_{1}^{\left(\mathrm{TOA}\right)}\rangle =\frac{2}{5}t_{0}+\frac{3}{5}t_{2}=\frac{22}{5}=4.4$ and the mean TOD is $\langle T_{1}^{\left(\mathrm{TOD}\right)}\rangle =t_{1}=4$. Also, the standard deviation of the TOA is $\sqrt{\frac{2}{5}t_{0}^{2}+\frac{3}{5}t_{2}^{2}-{\langle T_{1}^{\left(\mathrm{TOA}\right)}\rangle }^{2}}=\frac{4\sqrt{6}}{5}\approx 1.96$ while the standard deviation of the TOD is zero.

Now, we can also find the TF distribution to analyze the flow without separating it between TOA and TOD regions. In this case, the normalized TF distribution reads

$$\pi_{1}^{\left(\mathrm{TF}\right)}=\frac{1}{3}\delta \left(t-t_{0}\right)+\frac{1}{6}\delta \left(t-t_{1}\right)+\frac{1}{2}\delta \left(t-t_{2}\right).$$

Hence, we obtain that the mean TF, which quantify the average time-of-flow of the dynamics, is 13/3≈4.333 and the standard deviation of the TF is $\sqrt{29}/3\approx 1.795$.

As we see from above, the mean and standard deviation we obtained for the TOA, TOD, and TF are the one expected, showing that our approach is valid in these limiting cases.

## Appendix C. Proof of the Compact Form of Γ for the Three-Level Model

We define

(A9) $$\hat{\Gamma }\;\equiv \;\frac{i}{\hbar }\;\left[\hat{H},|2\rangle \langle 2|\right],$$

where the Λ-system Hamiltonian in the rotating-wave approximation is

(A10) $$\hat{H}\left(t\right)\;=\;\frac{\hbar \Omega_{1}}{2}\left(|1\rangle \langle 2|+|2\rangle \langle 1|\right)+\hbar \Delta \left(t\right)|2\rangle \langle 2|+\frac{\hbar \Omega_{2}}{2}\left(|3\rangle \langle 2|+|2\rangle \langle 3|\right).$$

Since the detuning term commutes with |2〉〈2|, it plays no role in the commutator. Using the general identity

$$\left[|a\rangle \langle b|,|2\rangle \langle 2|\right]=\delta_{b,2}|a\rangle \langle 2|-\delta_{a,2}|2\rangle \langle b|,$$

we obtain

(A11) $$\begin{array}{cc} \hat{\Gamma }\; & =-\frac{\Omega_{1}}{2}\left(-i\;|1\rangle \langle 2|+i\;|2\rangle \langle 1|\right)-\frac{\Omega_{2}}{2}\left(-i\;|3\rangle \langle 2|+i\;|2\rangle \langle 3|\right)\\ \; & =-\frac{\Omega_{1}}{2}\;{\hat{\sigma }}_{y}^{\left(1,2\right)}\;-\;\frac{\Omega_{2}}{2}\;{\hat{\sigma }}_{y}^{\left(3,2\right)}, \end{array}$$

where we introduced the Hermitian operators

$${\hat{\sigma }}_{y}^{\left(a,b\right)}\;\equiv \;-i|a\rangle \langle b|+i|b\rangle \langle a|.$$

Next, we define the *bright* and *dark* states

(A12) $$|B\rangle =\frac{\Omega_{1}|1\rangle +\Omega_{2}|3\rangle }{\Omega_{\mathrm{eff}}},\;|D\rangle =\frac{\Omega_{2}|1\rangle -\Omega_{1}|3\rangle }{\Omega_{\mathrm{eff}}},\;\Omega_{\mathrm{eff}}=\sqrt{\Omega_{1}^{2}+\Omega_{2}^{2}}.$$

In this basis one finds

(A13) $${\hat{\sigma }}_{y}^{\left(B,2\right)}\;=\;-i|B\rangle \langle 2|+i|2\rangle \langle B|=\frac{\Omega_{1}}{\Omega_{\mathrm{eff}}}{\hat{\sigma }}_{y}^{\left(1,2\right)}+\frac{\Omega_{2}}{\Omega_{\mathrm{eff}}}{\hat{\sigma }}_{y}^{\left(3,2\right)}.$$

It follows immediately that

(A14) $$\hat{\Gamma }=-\frac{\Omega_{\mathrm{eff}}}{2}\;{\hat{\sigma }}_{y}^{\left(B,2\right)}.$$

Thus, the current-like operator Γ is Hermitian and constant in time (for fixed couplings $\Omega_{1},\Omega_{2}$), acts only on the {|B〉,|2〉} subspace, and leaves the dark state |D〉 decoupled.

## Appendix D. TF–QSL Bound for the Hadamard Dephasing Model

We have

(A15) $$\begin{array}{cc} L^{†}\left({\hat{M}}_{+}\right) & =\frac{i}{\hbar }\left[\hat{H},{\hat{M}}_{+}\right]+\frac{\gamma }{2}\left({\hat{\sigma }}_{z}{\hat{M}}_{+}{\hat{\sigma }}_{z}-{\hat{M}}_{+}\right)\\ \; & =-\frac{\omega_{0}}{2\sqrt{2}}{\hat{\sigma }}_{y}-\frac{\gamma }{2}{\hat{\sigma }}_{x}. \end{array}$$

Hence,

(A16) $${\left(L^{†}\left({\hat{M}}_{+}\right)\right)}^{2}=\left(\frac{\omega_{0}^{2}}{8}+\frac{\gamma^{2}}{4}\right)\hat{I},$$

as $\{{\hat{\sigma }}_{y},{\hat{\sigma }}_{z}\}=0$ and ${\hat{\sigma }}_{x}^{2}={\hat{\sigma }}_{y}^{2}={\hat{\sigma }}_{z}^{2}=\hat{I}$. Hence, we obtain

(A17) $$\mathrm{Tr}\left[{\left(L^{†}\left({\hat{M}}_{+}\right)\right)}^{2}\right]=\frac{\omega_{0}^{2}}{4}+\frac{\gamma^{2}}{2}.$$

Combining Equations (A17), (23) and (26), we obtain Equation (38).
